# Phenotypic characterization of ESBL producing Enterobacter cloacae among children

**DOI:** 10.12669/pjms.291.2385

**Published:** 2013

**Authors:** Hafsa Amin, Aizza Zafar, Hasan Ejaz, Noor-ul-Ain Jameel

**Affiliations:** 1 Hafsa Amin, (M.Phil), Institute of Molecular Biology and Biotechnology, The University of Lahore, Lahore, Pakistan.; 2 Aizza Zafar, (M.Phil), Department of Microbiology, The Children’s Hospital and Institute of Child Health, Lahore, Pakistan.; 3 Hasan Ejaz, (M.Phil), Department of Microbiology, The Children’s Hospital and Institute of Child Health, Lahore, Pakistan.; 4 Noor-ul-Ain Jameel, (M.Phil), Institute of Molecular Biology and Biotechnology, The University of Lahore, Lahore, Pakistan.

**Keywords:** ESBL producing *Enterobacter* cloacae, Characterization of ESBL, Double-disk synergy test (DDST), CLSI confirmatory test

## Abstract

***Objective:*** The emergence of ESBL producing *Enterobacter cloacae* in clinical isolates is posing a serious threat for treating nosocomial infections. The aim of the study was to determine the frequency of extended spectrum β-lactamase (ESBL) producing *Enterobacter cloacae *and to compare the phenotypic methods used for the characterization of ESBL producing strains.

***Methodology:*** This cross sectional observational study was conducted during April 2011 to March 2012 at Microbiology department of The Children’s Hospital and Institute of Child Health, Lahore. A total number of 20,257 various clinical samples were analyzed during the study period. *Enterobacter cloacae *were identified using API 20E system and ESBL detection was carried out using double-disk synergy test (DDST) and CLSI confirmatory test.

***Results:*** *Enterobacter cloacae* were isolated from 221 samples, out of which 33 (14.93%) were ESBL producers and 188 (85.07%) were non-ESBL producers. The gender distribution of ESBL producing *Enterobacter cloacae *was 21 (63.6%) in males and 12 (36.4%) in females. Highest frequency (63%) of ESBL producing *Enterobacter cloacae* was detected in blood samples. Comparison of DDST and CLSI confirmatory test showed that 25 (75.75%) isolates were characterized by DDST and 33 (100%) using CLSI confirmatory test.

***Conclusion:*** The present study shows moderately high frequency of ESBL producing *Enterobacter cloacae *among children. DDST was found to be less efficient in ESBL detection as compared to CLSI confirmatory test.

## Introduction

 Extended-spectrum β-lactamases (ESBLs) are plasmid encoded enzymes that hydrolyze β-lactam ring and cause resistance to β-lactam antibiotics which include third-generation cephalosporins such as ceftriaxone, ceftazidime, cefotaxime and the monobactam such as aztreonam.^1^ The most common ESBLs are derived from widespread broad-spectrum β-lactamases TEM and SHV. Bacterial strains expressing these β-lactamases are presenting great therapeutic challenges. In recent years there has been a significant increase in incidence and prevalence of ESBL producing bacteria.^2^

 Nosocomial outbreaks of infections caused by ESBL-producing bacteria have been reported frequently.^3^ *Enterobacter cloacae*, has emerged as a major pathogen which causes nosocomial Gram-negative bloodstream infections.^4^ *Enterobacter cloacae* can be a serious cause of Gram-negative bacteremia resulting in nosocomial outbreaks in paediatric intensive care units (ICUs).^5^ Other infections include lower respiratory tract infections, skin and soft tissue infections, urinary tract infections, endocarditis, osteomyelitis and ophthalmic infections.^6^

*E. cloacae* has an inherent resistance to ampicillin and narrow-spectrum cephalosporins and exhibit a high frequency of mutation to resist expanded-spectrum cephalosporins.^7^^,^^8^ Carbapenems are generally used as treatment for multidrug-resistant organisms.^9^ The aim of the study was to evaluate the frequency of ESBL producing *E. cloacae* in hospitalized children and to compare the phenotypic characterization methods used for their detection to determine more accurate method.

## Methodology

 This cross sectional observational study was conducted at the Microbiology Department of The Children’s Hospital and Institute of Child Health Lahore, Pakistan, from April 2011 to March 2012. A total number of 20,257 pathological samples of blood, cerebro-spinal fluid, urine, sputum, peritoneal dialysis catheter, tracheal secretions and pus collected from various wards were analysed. The samples were cultured on solid media as Blood, Chocolate and MacConkey agar. Cystine Lysine Electrolyte Deficient Medium (CLED) was used only for urine culture samples. *Enterobacter cloacae *were identified by colonial morphology, Gram’s stain, catalase test, oxidase test and API 20E system (bioMerieux). A seven digit number generated on the basis of various biochemical reactions of API 20E system was checked by API 20E software to confirm *Enterobacter cloacae.*^10^

**Table-I:** Frequency distribution of ESBL producing *Enterobacter cloacae* from clinical samples in regards to gender.

*Site of isolation*	*No.*	*%*	*No. of AmpC producing E. cloacae*	
			*Males*	*Females*
Blood	21	63.6	12	9
Sputum	3	9	2	1
Urine	2	6	2	0
Wound swab	2	6	1	1
Pus	2	6	1	1
Cerebro-spinal fluid	1	3	1	0
Tracheal secretions	1	3	1	0
Peritoneal dialysis catheter	1	3	1	0
Total (33)			21	12

 A bacterial suspension of *Enterobacter cloacae* was made according to the 0.5 McFarland turbidity standard and an even lawn of bacteria was made on the Mueller Hinton agar petri plate (90mm). The screening for ESBL *E. cloacae *was performed using ceftazidime (30 μg) disk and ceftazidime resistant strains were considered as screen positives. DDST was performed by using disks containing amoxicillin/ clavulanate on Mueller-Hinton agar plate at a 20 mm distance from the indicator drugs; ceftazidime (30 μg) and cefotaxime (30 μg). ESBL production was seen by the clavulanate mediated enhancement of the activity of the indicator drug as a keyhole effect.^11^

 The CLSI confirmatory tests were performed using disks of ceftazidime (30 μg) and cefotaxime (30 μg) alone and in combination with ceftazidime-clavulanate (30/10 μg) and cefotaxime-clavulanate (30/10 μg). The CLSI confirmatory test was considered positive when the inhibition zone produced by the disks in combination clavulanate increased ≥5 mm than the disks without the clavulanate. The results of double disk diffusion test and CLSI test were compared.

## Results

*Enterobacter cloacae* were isolated from 221 culture positive samples, out of which 33 (14.93%) were ESBL producers and 188 (85.07%) were non-ESBL producers. The frequency of ESBL producing *Enterobacter cloacae* in male and female patients was 21 (63.6%) and 12 (36.4%), respectively. Occurrence of ESBL producing *Enterobacter cloacae* was found to be highest in the blood samples 21 (63.6%) (Table-I).

Comparison between DDST and CLSI confirmatory test showed that 25 (75.75%) isolates were identified by DDST and 33 (100%) using CLSI confirmatory test (Table-II).

## Discussion

 Extended spectrum β-lactamase (ESBL) producing *Enterobacter cloacae* is a rapidly emerging clinical pathogen which causes life threatening infections. This study provides the current data about the frequency and phenotypic characterization of ESBL producing *Enterobacter cloacae* isolated from different clinical samples of children.

**Table-II:** Comparison of DDST and CLSI for detection of ESBL (n= 33).

*Test*	*No. of positive isolates*	*(%)*
Double disk synergy test	25	75.75
CLSI confirmatory test	33	100

According to our study the frequency of ESBL producing *Enterobacter cloacae* was 14.93% among the culture positive samples. A study carried out in Microbiology department of Army Medical College, Rawalpindi reported a high frequency of 79% ESBL producing *E. cloacae *among clinical isolates recovered from Military Hospital, Rawalpindi.^12^ Similar research work conducted at The Aga Khan University Hospital, Karachi, Pakistan reported 50% ESBL positive *E. cloacae.*^13^ A study conducted in Lagos, Nigeria reported 37.5% ESBL producing *Enterobacter cloacae* among the clinical samples collected from two hospitals.^14^ Study work carried at University of Pittsburgh Medical Center (UPMC), Pennsylvania reported 33.33% frequency of ESBL producing *Enterobacter cloacae*.^15^ Improved hygienic patient care conditions and limited use of invasive devices could be the reason for low number of ESBL producing *Enterobacter cloacae* in our study when compared to the other studies.

 In our study, the frequency of ESBL producing *E. cloacae* was higher in males (63.6%) than females (36.4%). A study carried out at tertiary care hospital, Tanzania reported 41.2% in males and 58.8% in females.^16^ A research work conducted at University Hospital, northwest Spain revealed 65.8% ESBL producers in males and 34.2% in females.^17^ In another study conducted at a cardiothoracic intensive care unit, Spain reported 42.9% ESBL producing *E. cloacae* in males and 57.1% in females.^18^ The distribution pattern of ESBL producing *E. cloacae* varies in different studies suggesting that these infections are not gender specific and the ratio of male patients attending our hospital during the study period might be higher than the female patients.

 We found highest percentage of ESBL producing *Entrobacter cloacae* in the blood samples (63.6%). Strains were also recovered from sputum (9%), urine (6%) and wound swabs (6%). Aibinu et al conducted a study for the presence of ESBL producing *E. cloacae *in clinical isolates. They found the highest frequency in urine samples (30%) followed by respiratory 6 (15%) and Blood 4 (10%) specimens.^14^ A study conducted in Huashan Hospital, Shanghai reported higher number of positive isolates from sputum and urine (37.93% each).^19^ The percentage of ESBL producing *Enterobacter cloacae *in blood samples is much higher in our study as compared to other studies, which shows that ESBL producing *Enterobacter cloacae* caused bloodstream infections more frequently than urinary infections in our setup.

 The comparison of DDST and CLSI confirmatory test showed that the higher numbers of positive isolates were detected by CLSI confirmatory test (100%) than the DDST (75.75%). A study conducted by collecting 91 ESBL producers from 32 hospitals in Kinki area of Japan reported DDST positive for 97.80% of the isolates and was negative for only 2.19% of isolates.^20^ Rao et al used DDST and CLSI confirmatory test on 126 ESBL screen positive isolates. The DDST method detected 109 (86.5%) and the CLSI 93 (73.8%) cases.^21^ Study conducted by Dechen et al showed that ESBL producers can be detected by DDST and CLSI confirmatory test with equal efficacy. Their results showed 100% agreement in DDST and CLSI method for detection of ESBL producers.^22^ Another study from India reported 135 screen positive ESBL producers. The DDST showed positive results in 126 (93.3%) while CLSI in 135 (100%) cases.^23^ These studies support the results of our study where CLSI confirmatory test is found to be better than DDST.

 In conclusion, moderately high frequency of ESBL producing *Enterobacter cloacae *was found at our institute. CLSI confirmatory tests generated better results than DDST. Due to the wide spread of ESBL producing strains, it is important to maintain the active surveillance system at microbiological laboratories for early detection of ESBL producing organisms. Preventive measures to stop the colonel spread of the resistant strains could significantly reduce the risk of treatment failure and help in the generation of sound epidemiological data.

## References

[1] Kenneth S, Thomson KS, Sanders CC (1997). A simple and reliable method to screen isolates of Escherichia coli and Klebsiella pneumoniae for the production of TEM- and SHV-derived extended spectrum β-lactamases. Clin Microbiol Infect Dis.

[2] Bradford PA (2001). Extended-Spectrum β-Lactamases in the 21st Century: Characterization, Epidemiology, and Detection of This Important Resistance Threat. Clin Microbiol.

[3] Liu SC, Leu HS, Yen MY, Lee PI, Chou MC (2002). Study of an outbreak of Enterobacter cloacae sepsis in a neonatal intensive care unit: the application of epidemiologic chromosome profiling by pulsed-field gel electrophoresis. Am J Infect Control.

[4] Sanders WEJr, Sanders CC (1997). Enterobacter spp.: pathogens poised to flourish at the turn of the century. Clin Microbiol Rev.

[5] Falkiner FR (1992). Enterobacter in hospital. J Hosp Infect.

[6] Dijk YV, Bik EM, Vernooij SH (2002). Management of an outbreak of Enterobacter cloacae in a neonatal unit using simple preventive measures. J Hosp Infect.

[7] Then RL (1987). Ability of newer beta-lactam antibiotics to induce beta-lactamase production in Enterobacter cloacae. Eur J Clin Microbiol.

[8] Bush K, Jacoby GA (2010). Updated functional classification of β-lactamases. Antimicrob Agents Chemother.

[9] Zanetti G, Bally F, Greub G (2003). Cefepime versus imipenem-cilastatin for treatment of nosocomial pneumonia in intensive care unit patients a multicenter, evaluator-blind, prospective, randomized study. Antimicrob Agents Chemother.

[10] Cheesbrough M (2000). District laboratory practice in tropical countries (2).

[11] Clinical and Laboratory Standards Institute (CLSI) (2010). Performance standards for antimicrobial susceptibility tests.

[12] Ali AM, Rafi S, Qureshi AH (2004). Frequency of extended spectrum beta-lactamase producing gram negative bacilli among clinical isolates at clinical laboratories of Army Medical College, Rawalpindi. JAMC.

[13] Jabeen K, Zafar A, Hasan R (2005). Frequency and sensitivity pattern of extended spectrum beta-lactamase producing isolates in a tertiary care hospital laboratory of Pakistan. JPMA.

[14] Aibinu IE, Ohaegbulam VC, Adenipekun EA, Ogunsola FT, Odugbemi TO, Mee BJ (2003). Extended-Spectrum β-Lactamase Enzymes in Clinical Isolates of Enterobacter Species from Lagos, Nigeria. J Clin Microbiol.

[15] Szabo D, Bonomo RA, Silveira FA, Pasculle AW, Baxter C, Linden PK (2005). SHV-Type Extended-Spectrum Beta-Lactamase Production Is Associated with Reduced Cefepime Susceptibility in Enterobacter cloacae. J Clin Microbiol.

[16] Mshana SE, Gerwing L, Minde M, Hain T, Domann E, Lyamuya E (2011). Outbreak of a novel Enterobacter sp. carrying blaCTX-M-15 in a neonatal unit of a tertiary care hospital in Tanzania. Int J Antimicrob Agents.

[17] Fernandez A, Pereira MJ, Suarez JM, Poza M, Trevino M, Villalon P (2011). Emergence in Spain of a Multidrug-Resistant Enterobacter cloacae Clinical Isolate Producing SFO-1 Extended-Spectrum β-Lactamase. J Clin Microbiol.

[18] Manzur A, Tubau F, Pujol M, Calatayud L, Domingue MA, Pena C (2007). Nosocomial Outbreak Due to Extended-Spectrum-Beta-Lactamase- Producing Enterobacter cloacae in a Cardiothoracic Intensive Care Unit. J Clin Microbiol.

[19] Jiang X, Ni Y, Jiang Y, Yuan F, Han L, Li M (2005). Outbreak of Infection Caused by Enterobacter cloacae Producing the Novel VEB-3 Beta-Lactamase in China. J Clin Microbiol.

[20] Komatsu M, Ajhara M, Shimakawa K, Iwasaki M, Nasgasaka Y, Fukuda S (2003). Evaluation of MicroScan ESBL confirmation panel for Enterobacteriaceae producing, extended-spectrum β-lactamases isolated in Japan. J Diagn Micr Infec Dis.

[21] Rao SPN, Basavarajappa KG, Krishna GL (2008). Detection of extended spectrum beta-lactamase from clinical isolates in Davangere. Indian J Pathol Microbiol.

[22] Dechen CT, Shyamasree D, Luna A, Ranabir P, Takhellambam SKS (2009). Extended Spectrum Beta-lactamase Detection in Gram-negative Bacilli of Nosocomial Origin. J Glob Infect Dis.

[23] Gaurav D (2012). Prevalence of Extended Spectrum Beta Lactamase (ESBL) Producers among Gram Negative Bacilli from Various Clinical Isolates in a Tertiary Care Hospital at Jhalawar, Rajasthan, India. JCDR.

